# The Generation and Characterization of a Mouse Embryonic Stem Cell Line with Psmb9 Immunoproteasome Gene Knockout

**DOI:** 10.32607/actanaturae.27583

**Published:** 2025

**Authors:** D. V. Kriger, U. I. Podenkova, A. A. Kuzmin, N. D. Aksenov, A. V. Kropacheva, A. S. Zinovyeva, A. V. Selenina, A. N. Tomilin, A. S. Tsimokha

**Affiliations:** Institute of Cytology, Russian Academy of Sciences, St-Petersburg, 194064 Russia

**Keywords:** Psmb9, Lmp2, immunoproteasome, mouse ESCs, differentiation

## Abstract

Immunoproteasomes, a unique type of proteasome complex, play a critical role in antigen presentation and cellular homeostasis. Unlike the constitutive 20S proteasome, the catalytic subunits β1, β2, and β5 in the immunoproteasome are replaced by inducible isoforms: β1i (LMP2), β2i (MECL-1), and β5i (LMP7). The expression of the genes encoding these subunits (Psmb9, Psmb10, and Psmb8) is activated by cytokines, primarily interferon-γ (IFNγ). Although it has been demonstrated more and more convincingly that immunoproteasomes are expressed in embryonic stem cells (ESCs), their involvement in maintaining pluripotency, promoting self-renewal, and regulating differentiation processes remains unexplored. This study implemented CRISPR/Cas9 technology to generate a *Psmb9 *gene knockout (Psmb9KO) mouse ESC line. The resulting cells exhibited a normal karyotype and morphology, maintained normal proliferation rates, and retained the capacity to form teratomas containing derivatives of all three germ layers. However, the differentiation induced by retinoic acid (RA) and IFNγ caused an accumulation of Mecl-1 precursors in Psmb9KO cells, suggesting modifications in immunoproteasome assembly. Furthermore, an increase in the caspase-like activity of immunoproteasomes was detected, suggesting the integration of a constitutive β1-subunit into the complex in place of Lmp2. The findings underscore the adaptability of the ubiquitin-proteasome system in maintaining cellular proteostasis by compensatory mechanisms that counteract the lack of Lmp2. The Psmb9KO line can serve as a valuable model for examining the function of immunoproteasomes in proteostasis regulation during early mammalian embryogenesis differentiation.

## INTRODUCTION

For the body to function properly and maintain its integrity, proteins must circulate within cells without interruption. The cellular protein equilibrium – known as proteostasis – is maintained through the concerted activity of the *de novo *protein synthesis machinery and the degradation processes responsible for the removal of damaged or superfluous proteins. Cellular protein degradation is mainly mediated by autophagy and the ubiquitin-proteasome system (UPS). At least 80% of intracellular proteins are degraded by the UPS [1]. This particular system identifies and directs ubiquitinated proteins for proteasomal degradation.

proteasome [3]. Proteolytic cleavage of protein substrates within the proteasome happens in a “catalytic” chamber, which is formed by two central β-rings. In eukaryotes, three subunits, commonly referred to as β1, β2, and β5, perform proteolytic activity. These catalytic subunits are known to possess differences in their substrate specificity. The β1 subunit can cleave peptide bonds following “acidic” amino acid residues, a process referred to as caspase-like activity. The β2 subunit exhibits trypsin-like activity and cleaves polypeptide chains subsequent to basic amino acids. As for the β5-subunit, it is characterized by chymotrypsin- like activity, which leads to the hydrolysis of peptide bonds following hydrophobic amino acid residues. The catalytic chamber is completely sealed from the external environment, preventing accidental degradation of proteins and ensuring process specificity. The N-terminal sequences of α-subunits act as a gate, limiting the access of substrate proteins to the catalytic chamber of the core particle, opening only upon binding to the regulatory particle [4], thereby ensuring stringent regulation of the protein degradation process.

Upon stimulation of mammalian cells with interferon-γ (IFNγ), the expression of alternative catalytic subunits is induced, resulting in the assembly of a modified 20S proteasome, also known as the immunoproteasome [5]. Within the immunoproteasome, the constitutive catalytic β-subunits (β1, β2, and β5) are replaced by their inducible counterparts: Lmp2 (β1i), Mecl-1 (β2i), and Lmp7 (β5i). These substitutions alter the proteolytic activity of the proteasome complex [5, 6], as the Lmp2 (β1i) and Lmp7 (β5i) subunits exhibit chymotrypsin-like activity, whereas Mecl-1 (β2i) is defined by trypsin-like activity. Immunoproteasomes are crucial in the generation of peptide antigens for MHC I presentation [7], by which they play a vital role in antiviral [8] and antitumor defense [9]. In addition to their role in antigen presentation, immunoproteasomes play a crucial role in the regulation of proteostasis by inhibiting the accumulation of damaged or misfolded proteins within the cell [10, 11]. Furthermore, during the differentiation of human embryonic stem cells (ESCs), immunoproteasome activity gradually recedes, suggesting their involvement in adaptive shifts in the cellular state [12]. In addition, immunoproteasomes have been shown to participate in the degradation of oxidized proteins, which plays a crucial role in the maintenance of proteome integrity under conditions of cellular differentiation-induced stress. For example, in mouse ESCs, immunoproteasomes are activated through the accumulation of oxidized proteins, but this activity increases substantially during differentiation, not in the state of pluripotency [13, 14]. According to our research, mouse ESCs activate the expression of all three catalytic subunits of the immunoproteasome when transitioning out of the naïve pluripotency state [15]. Consequently, recent data suggest that the role of immunoproteasomes in pluripotent cells may be associated with preparation for differentiation, thereby facilitating the degradation of damaged proteins and maintaining cellular homeostasis. Nonetheless, the functional role of immunoproteasomes in maintaining pluripotency and self-renewal in ESCs remains for the most part unexplored.

Previously, we generated and characterized a mouse embryonic stem cell line with knockout of the *Psmb8 *gene, which encodes the immunoproteasome subunit Lmp7 (β5i) [16]. To examine the contributions of individual catalytic subunits of the immunoproteasome during early embryogenesis, we acquired mouse ESCs with *Psmb9 *gene knockout, which encodes another catalytic subunit of the immunoproteasome: Lmp2 (β1i). The resulting cell lines underwent genotyping, karyotyping, and functional characterization, encompassing the assessment of proliferation rates, analysis of pluripotency marker expression, determination of the Lmp7 and Mecl-1 expression levels, evaluation of proteasome proteolytic activities, and assessment of their capacity for* in vivo *differentiation.

## EXPERIMENTAL PART

**Cell culture**

This study used mouse ESCs of the E14 Tg2a line. The cells were cultivated at 37°C in a humidified atmosphere with 5% CO_2_. Culture dishes were pre-coated with a 0.1% gelatin solution (Sigma, USA). The SL medium based on Knockout DMEM (Thermo Fisher, USA) was supplemented with 15% heat-inactivated fetal bovine serum (HyClone, GE Healthcare Life Sciences, UK), 100 U/mL penicillin and 100 μg/mL streptomycin (Thermo Fisher, USA), 2 mM L-glutamine (Thermo Fisher, USA), a 100 μM non-essential amino acids solution (Thermo Fisher, USA), 100 μM β-mercaptoethanol (Sigma, Germany), and a 500 U/mL leukemia inhibitory factor (LIF, produced in our laboratory).

The protein levels of the immunoproteasome subunits were evaluated after ESCs had been differentiating for 2 days in the SL medium without LIF but supplemented with 0.1 μM retinoic acid (RA, Sigma). Subsequently, the cells remained in culture for an additional 24 h within a medium enriched with IFNγ (ProSpec, Israel) at a concentration of 150 U/mL.

**Generation of *Psmb9 *knockout mouse ESCs**

The Psmb9 gene was inactivated utilizing CRISPR/Cas9 genome editing technology. The guide RNA (gRNA) sequence (5′-GTTTGACGGGGGTGTCGTGG- 3′) was selected using the online Benchling tool (https://www.benchling.com). This sequence was subsequently cloned into the pX330-U6-Chimeric_BB-CBh-hSpCas9 vector (Addgene, USA), which contains the gene for the green fluorescent protein (GFP). A control ESC line was generated by transfecting the cells with nonspecific gRNA (Scrambled) [17]. Transfection was performed using the FuGene HD reagent (Promega, USA) according to the manufacturer’s protocol. Selection of the transfected cells was performed by fluorescence-activated cell sorting (FACS) using an S3e Cell Sorter (Bio-Rad Lab., USA). The cells were prepared according to a previously described procedure [16]. The sorted cells were plated at low density and cultured for 10–14 days. The selected clones were checked by immunoblotting with anti-Lmp2 antibodies and by sequencing.

**Genomic DNA extraction and sequencing**

Genomic DNA was isolated according to a protocol described previously [17]. The regions of the flanks of the gRNA target site were amplified by PCR using the primers 5′-AACTGCAGATAACACAGTCCATC-3′ and 5′-CCAGGACCAGGAAAGACCTGG-3′. The products were cloned into the pAL2-T vector (Eurogen, Russia). Subsequent sequencing (Eurogen, Russia) was performed using the universal M13 primer.

**Table 1:** gRNA sequences and possible off-target sites with primers designed for amplifying specific genomic regions

Type	Sequence (5’ -> 3’	Score	Cleavage-prone chromosomal region	Primers (5’ -> 3’)
gRNA	GTTTGACGGGGGTGTCGTGG	100	chr17:-34404735	AACTGCAGATAACACAGTCCATC CCAGGACCAGGAAAGACCTGG
Off-target 1	GTGTGAAGGGGGTGTCATGG	0.9	chr7:+15781982	AAGTGCAGGTCCTCTGAAAAGAA AGAAATGGAGTAGTGTGCTCCACAA
Off-target 2	AGTAGACGGGGGTGTCGTGC	0.9	chr16:+96466310	CTCTTGTCTTCCTCTCCCTGT GCTTGGACCCTAGAGTGGAA
Off-target 3	GTCAGACTGGGGTGTCCTGG	0.7	chr6:+28141384	TCGGATCTAGGAAGCAGTCTC GCAGTAGATAGCCTGAACCTG
Off-target 4	TTATGACGTGAGTGTCGTGG	0.6	chr14:-118326405	AGTCTGGTCTAGAGCTGTCCTC TCCTTTGGGAGTAGGGCTATGT
Off-target 5	GCTGGATGGGGGTGTCTTGG	0.5	chr5:+114566059	ATAAACGGCCAAGGTCAACC TGGGAGACACAGATTCCTAAACT

Potential off-target sites for the selected gRNA were searched for using the online Benchling tool. The genomic regions encompassing these potential off-target sites were amplified by PCR with specific primers (*Table 1*) and sequenced (Eurogen, Russia).

**Karyotyping**

Metaphase spreads were prepared following a procedure described previously [18]. The microscopic analysis of the preparations was performed using an EVOS FL Auto Imaging System (Applied Biosystems, USA) with immersion oil at ×100 magnification. The chromosomes were counted using Fiji (ImageJ) software. A cell line was considered normal if the sample contained more than 90% of cells with the standard mouse chromosome number of 40.

**Determination of ESC proliferative activity**

The proliferative activity of the control (Scr) and Psmb9 knockout (Psmb9KO) cell lines was assessed on the third day after seeding. The cells were passaged at a concentration of 5 × 10³ live cells/cm². Prior to their counting, the cells were trypsinized with a 0.05% trypsin/EDTA solution (Gibco, USA) and pelleted. The pellet was then resuspended in PBS containing 50 μg/ml propidium iodide (PI). Cell counting was performed on a Coulter EPICS XL Flow Cytometer (Beckman Coulter, USA).

**Immunocytochemical staining and microscopy**

Immunocytochemical staining was performed according to a procedure described previously [19]. Image acquisition and subsequent analysis were performed using a CellVoyager CQ1 high-content screening system (Yokogawa Electric, Japan). The primary antibodies used were Nanog (1 : 500, Bethyl A300-397), Oct4 (1 : 300, Santa Cruz sc-5279) and secondary antibodies conjugated to Alexa 488 and 568 fluorophores (a-11008 and a-11004), respectively (Invitrogen, USA).

**Immunoblotting**

Cell extracts were obtained using a method described previously [15]. Each sample was separated by electrophoresis on a 13% SDS-polyacrylamide gel, with proteins transferred onto a 0.45 μm PVDF membrane in a Tris-glycine buffer (Bio-Rad, USA). After the transfer, the membrane was incubated in a solution of 5% non-fat dry milk in PBS buffer. For protein visualization, the membrane was incubated overnight at 4°C with specific primary antibodies, followed by a 1 h incubation with the appropriate secondary antibodies. Chemiluminescence was detected using a ChemiDoc imaging system (Bio- Rad, USA). The study employed the following primary antibodies: Lmp2 (1 : 500, Abclonal A9549), Lmp7 (1 : 5000, kindly provided by Prof. Dr. Ulrike Seifert, University Medicine Greifswald, Germany), Mecl-1 (1 : 500, Abcam ab183506), Oct4 (1 : 500, Santa Cruz sc-5279), Nanog (1 : 500, Cell Signaling #8822), α7 (1 : 1000, Enzo Life Sciences PW8110), β2 (1 : 1000, Enzo Life Sciences PW9300), β5 (1 : 1000, Bethyl A303-847), Rpn1 (1 : 1000, ServiceBio GB113525), Rpt2 (1 : 1000, ServiceBio GB114427), and β-Actin (1 : 5000, Cell Signaling #3700). Additionally, we used secondary antibodies conjugated to horseradish peroxidase (HRP): against rabbit IgG (1 : 5000, Jackson ImmunoResearch, 111-035-003) and against mouse IgG (1 : 5000, Jackson ImmunoResearch, 115-035-062).

**Determining the proteasomal proteolytic activity**

The caspase-like (CL) peptidase activity of the proteasomes in the cell extracts (~6 μg) was measured using the substrate Z-LLE-AMC (carbobenzoxy- Leu-Leu-Glu-7-amido-4-methylcoumarin) (Enzo Life Sciences, Germany) at a concentration of 0.25 mM. The reaction was conducted in a buffer containing 50 mM Tris-HCl (pH 7.5), 5 mM MgCl_2_, 40 mM KCl, 1 mM DTT, and 1 mM ATP, at 37°C for 30 minutes according to a previously described procedure [20]. The reaction was stopped by adding an equal volume of 2% SDS. The proteasomal activity was determined by measuring the fluorescence of free AMC (7-amino- 4-methylcoumarin) using a VersaFluor fluorometer (Bio-Rad, USA) at excitation and emission wavelengths of 340–380 nm and 455–465 nm, respectively. The values obtained were normalized by subtracting the background fluorescence and recalculated using an equalization coefficient (determined after measuring the protein concentration in the samples). Finally, the mean values and standard deviations were calculated.

**Teratoma assay**

All the experiments were performed on BALB/c mice obtained from the Lobachevsky University Laboratory Animal Breeding Facility (Nizhny Novgorod, Russia). The animals were housed in individual cages under a 12-hour light/dark cycle in a temperature-controlled room (22°C) with ad libitum access to food and water. All the studies were conducted in accordance with the principles of biomedical ethics outlined in the 1964 Helsinki Declaration and its later amendments and were approved by the Commission on Biological Safety and Bioethics of the Institute of Cytology of the Russian Academy of Sciences (St. Petersburg, Russia), protocol No. 02/24 dated June 6, 2024. The resulting ESCs (2 × 10^6^ cells) were injected subcutaneously into the hind limbs of the mice to assess their pluripotent properties. Four weeks after injection, the mice were euthanized. The resulting teratomas were excised, weighed, fixed in Bouin’s solution (75 ml saturated aqueous picric acid solution; 25 ml 40% aqueous formaldehyde solution; 5 ml glacial acetic acid), and embedded in paraffin. Sections 7 μm thick were prepared from the paraffin blocks, deparaffinized, stained with hematoxylin and eosin (BioVitrum, Russia), and examined microscopically using an EVOS FL Auto Imaging System (Applied Biosystems, USA).

**Statistical analysis**

All the immunofluorescence and immunoblotting images presented in this work emanate from at least three independent experiments. Flow cytometry analysis was performed on a minimum of 1 × 10^4^ cells per sample. The data are presented as the mean value ± standard deviation (SD) from a minimum of three replicates. Statistical significance was determined at a level of *p* < 0.05 using a one-way analysis of variance (ANOVA) performed with the GraphPad Prism 8 software.

## RESULTS

**Fig. 1:**
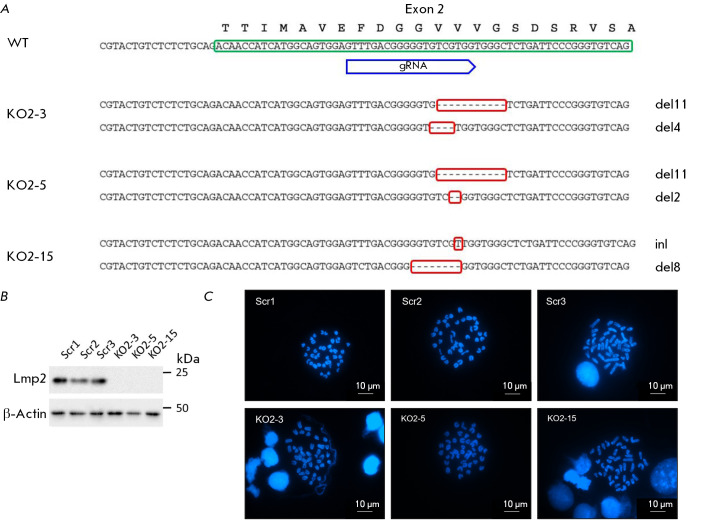
Validation of Psmb9 gene knockout in mESCs. (A) Genotyping results of three mESC lines with Psmb9 gene knockout (KO2-3, KO2-5, KO2-15). WT – sequence of the second exon of the Psmb9 gene in wild-type mESCs. Indel mutations are highlighted with a red rectangle, del – deletion, in – insertion. (B) Western blot analysis of Lmp2 protein expression levels in the control (left) and Psmb9KO clones (right) following differentiation induced by RA and IFNγ treatment (see “Experimental Part”). β-Actin served as a loading control. (C) Representative images of metaphase spreads from the control lines (Scr) and Psmb9KO cells; the chromosomes were stained with DAPI. The scale bar is 10 μm

**Fig. 2:**
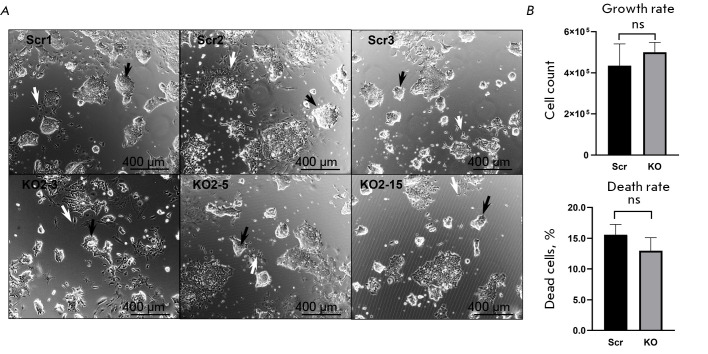
Psmb9 knockout does not affect the morphological and proliferative characteristics of mESCs. (A) Representative images of Psmb9KO mESCs and control lines (Scr1–3) under standard culture conditions. Colonies with a morphology characteristic of undifferentiated mESCs are marked with black arrows. Cells that undergo spontaneous differentiation are indicated by white arrows. The scale bar is 400 μm. (B) Comparison of the cellular proliferation rates between Psmb9KO lines and control lines (Scr). Cell death analysis was performed using propidium iodide (PI) staining. Data are presented as the mean ± standard deviation (n = 3). ns – not statistically significant (one-way ANOVA)

ESC lines with knockout of the *Psmb9 *gene (Psmb9KO) were obtained using the CRISPR/Cas9 genomic editing system. The gRNA targeting the second exon of the *Psmb9 *gene was cloned into the pX330-U6-Chimeric-BB-CBh-hSp-Cas9 plasmid. This plasmid also encodes the green fluorescent protein (GFP). The construct was introduced into the cells through transfection, followed by the selection of GFP-positive cells via fluorescence-activated cell sorting (FACS) (see the Experimental Part). As a result, more than 20 clones lacking the Lmp2 protein were identified using immunoblotting data. Among these, clones containing indels (insertions and/or deletions) in both alleles of the *Psmb9 *gene were identified by TA-cloning and sequencing (*Fig. 1A*). As expected, these mutations caused a frameshift, leading to the disruption of functional Lmp2 protein translation (*Fig. 1B*). The five most probable potential off-target sites (*Table 1*) which could have been modified due to nonspecific interaction with the gRNA were assessed in the selected Psmb9KO ESC clones. Furthermore, all selected Psmb9KO ESC clones were confirmed by chromosome counting to contain 40 chromosomes (*Fig. 1C*), corresponding to the normal karyotype of mouse ESCs. The morphology of the KO_2_-3, KO_2_-5, and KO_2_-15 cell lines cultured on gelatin-coated plastic substrates did not exhibit significant differences from that of the control cells (Scr) (*Fig. 2A*). Additionally, the proliferation and cell death rates *in vitro *for all three lines were comparable to those of the control lines (*Fig. 2B*).

**Fig. 3:**
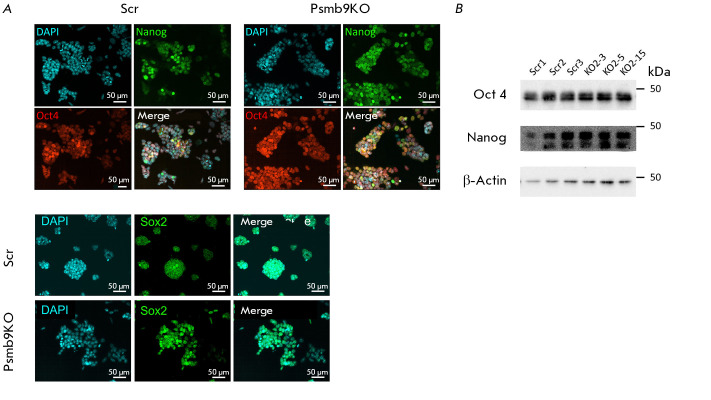
Loss of Psmb9 expression does not affect the expression of pluripotency markers. (A) Immunocytochemistry staining of the pluripotency markers Nanog, Oct4, and Sox2; representative images of the control cells (Scr) and Psmb9KO cells are presented. The nuclei were stained with DAPI. The scale bar is 50 μm. (B) Western blot analysis of Nanog and Oct4 expression in Scr lines and Psmb9KO cells cultured in SL media. β-Actin was used as a loading control

The staining intensity of the pluripotency factors Nanog, Oct4, and Sox2, as determined by immunocytochemical analysis, remained consistent, irrespective of *Psmb9* gene expression levels (*Fig. 3A*). Additionally, the immunoblotting data demonstrated that the levels of the Nanog and Oct4 factors remained unchanged despite the lack of the Lmp2 protein (*Fig. 3B*).

**Fig. 4:**
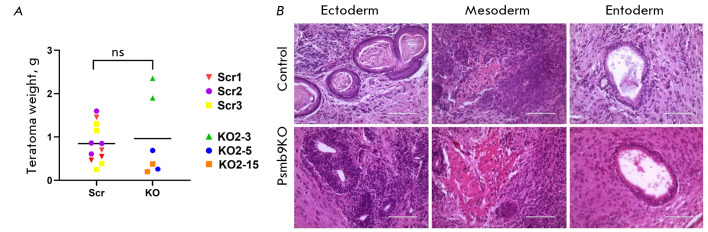
Psmb9KO ESC lines retain pluripotency, giving rise to derivatives of all three germ layers. (A) Dot plot showing individual teratoma masses derived from the control (Scr) and Psmb9KO mESC lines. Horizontal lines represent the mean teratoma mass for each group. ns – not statistically significant (one-way ANOVA). (B) Histological analysis of teratomas formed by Psmb9KO ESCs. The teratomas contain cell structures representative of all three germ layers: ectoderm (keratinizing epithelium and neuroepithelial rosettes), mesoderm (chondroblasts and chondrocytes in mesenchyme), and endoderm (differentiating enterodermal epithelium). Samples were counterstained with hematoxylin and eosin. The scale bar is 100 μm

Next, we assessed the ability of Psmb9KO cells to differentiate *in vivo *within teratomas. The sizes of the teratomas formed by Psmb9KO cells were similar to those formed by the control cells (*Table 2*, *Fig. 4A*). Both the control and Psmb9KO cells were demonstrated using histological analysis to successfully differentiate into derivatives of all three germ layers: areas of keratinized epithelium and neuroepithelial rosettes (ectoderm), cartilage (mesoderm), and ciliated epithelium (endoderm) (*Fig. 4B*). Thus, we had successfully generated a panel of mouse ESC lines with *Psmb9 *knockout. The panel is expected to be invaluable for further research.

**Fig. 5:**
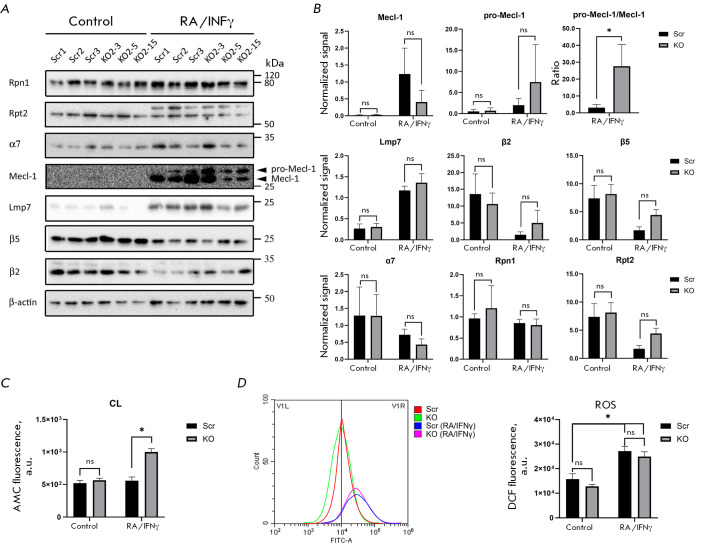
Analysis of proteasome activity and reactive oxygen species (ROS) production in Psmb9 knockout mESCs. The control cell lines (Scr1-3) and Psmb9KO mESCs (KO2-3, 2-5, and 2-1) were cultured in a SL medium. Differentiation was induced with retinoic acid (RA) for 2 days, followed by IFNγ treatment for 1 day. (A) Western blot analysis of constitutive and immunoproteasome subunit expression in Psmb9 knockout mESCs compared to the control cells (Scr). β-Actin was used as a loading control. (B) Integral intensity measurements of the western blot bands shown in (A), normalized to β-actin. (C) Measurement of the caspase-like (CL) activity of the 20S proteasome. (D) Assessment of ROS production. Representative histograms of cell distribution by fluorescence intensity in the FITC channel are shown. The rightward shift of the histogram indicates increased ROS production. Data are presented as mean ± standard deviation (n = 3). ns – not statistically significant; *p < 0.05 (one-way ANOVA)

In Psmb9KO ESCs, the immunoblot analysis of proteasomal protein expression revealed no significant changes in the levels of the α7, β2, and β5 subunits of the 20S proteasome (*Fig. 5A,B*). Furthermore, the protein levels of the 19S regulatory subunits (Rpn1 and Rpt2) remained constant, suggesting that the fundamental structure of the proteasome complex remains undisturbed in the absence of a *Psmb9 *gene product. No significant change in the expression of the immunoproteasome subunits Lmp7 (β5i) and Mecl-1 (β2i) was recorded between the Psmb9KO and control ESCs. At the same time, retinoic acid (RA)-differentiated and interferon-γ (IFNγ)-treated Psmb9KO cells tended to exhibit a lower content of the mature form of the immunoproteasome subunit Mecl-1 (β2i) while accumulating its non-processed form (pro-Mecl-1) compared to the control ESCs. However, these differences did not attain statistical significance (*Fig. 5A,B*). That notwithstanding, the ratio of signal levels between the precursor form of pro-Mecl-1 and the mature form of Mecl-1 indicated that the precursor accumulated more significantly in Psmb9-knockout cells than it did in the controls (*Fig. 5A,B*). The absence of specific antibodies was an impediment to us evaluating the levels of the β1 catalytic subunit in Psmb9KO cells. For this reason, we examined the peptidase activity of the 20S proteasome in cell extracts employing a fluorogenic substrate specific to this subunit. Following RA-induced differentiation and IFNγ treatment, Caspase-like activity was found to be significantly enhanced in Psmb9KO ESCs compared to the control cells, indicating an upregulation of the β1 subunit in Psmb9KO cells and its incorporation into the 20S proteasome instead of Lmp2, β1i (*Fig. 5C*).

Competing studies suggest that the absence of Lmp2 in mouse and rat cells can lead to oxidative stress through the accumulation of reactive oxygen species (ROS) [21, 22]. We conducted a comparative analysis of ROS production in the *Psmb9* knockout cell versus the control cell lines. Following RA-induced differentiation and IFNγ treatment, an increase in ROS levels was detected across all the cells under analysis. However, we found no differences in ROS production associated with *Psmb9 *expression (*Fig. 5D*).

**Table 2:** Teratoma mass values from individual mice transplanted with control (Scrambled) or Psmb9 knockout (Psmb9KO) embryonic stem cells (ESCs)

Group	ESC type	Number of animals (n)	Individual values of teratoma masses, g
Control	Scrambled #1	4	1.45, 0.7, 0.55, 0.47
	Scrambled #2	4	0.85, 1.6, 0.86, 0.61
	Scrambled #3	4	1.3, 1.15, 0.39, 0.25
Psmb9 KO	KO 2-3	2	1.9, 2.35
	KO 2-5	2	0.69, 0.26
	KO 2-15	2	0.38, 0.20

## DISCUSSION

The immunoproteasome subunit Lmp2 (β1i) encoded by the *Psmb9 *gene has been reported to play a crucial role in numerous processes related to immune defense [23, 24, 25, 26], maintenance of cellular homeostasis [10, 21, 27, 28], and tissue development [22, 29]. Furthermore, the expression of *Psmb9*, along with other immunoproteasome subunits, has been demonstrated to be essential during the early stages of mammalian embryogenesis [12]. Given the diverse functions of Lmp2 in human cells, investigating its mechanisms of action is of significant interest. In the present study, we generated a cell model of mouse ESCs with *Psmb9 *gene knockout using the CRISPR/Cas9 technology. The resulting lines exhibited no impairments in the growth rate or expression levels of key pluripotency markers compared to the control lines. Moreover, they successfully formed teratomas in immunodeficient mice. The histological analysis of teratomas revealed structures derived from all three embryonic germ layers, thus confirming the retention of the pluripotent potential in *Psmb9*-deficient ESCs. The absence of Psmb9 expression did not alter the protein levels of the proteasomal subunits α7 and the catalytic β2 and β5 or the levels of the other two immunoproteasome subunits, Lmp7 (β5i) and Mecl-1 (β2i). However, RA-differentiated and IFNγ Psmb9KO-treated ESCs were observed to accumulate the precursor form of Mecl-1. The data suggest that in mouse ESCs, immunoproteasomes lacking the Lmp2 subunit can still exist as an intermediate, albeit with less efficient assembly, which aligns with prior studies [23]. Furthermore, the differentiation of Psmb9KO ESCs resulted in an elevation of caspaselike activity in the 20S proteasome. This suggests a possible compensatory integration of the β1-subunit into the immunoproteasome complex, substituting Lmp2. This substitution mechanism may be interpreted as an adaptive response to the absence of Lmp2, which maintains functional UPS activity in IFNγ- induced differentiated cells.

The *Psmb9 *and *Psmb8 *genes, which encode the Lmp2 and Lmp7 subunits, respectively, are located within the major histocompatibility complex class II (MHC II) locus [30]. The part played by these subunits in the regulation of the immune response have been a subject of considerable research since they were first described. Impaired Lmp2 function was reported to disrupt antigen presentation [24], alter the repertoire of CD8^+^ T lymphocytes [26], and cause an absolute collapse in their numbers in mice [25]. Immunoproteasomes, including Lmp2, are involved in the maintenance of proteostasis and the regulation of cell differentiation. Increased Lmp2 levels are observed under cellular stress conditions, such as mitochondrial dysfunction in human cells, which results in elevated proteasome activity and reduced accumulation of oxidized proteins [27]. Additionally, Lmp2 loss is correlated with the onset of neurodegenerative changes in Alzheimer’s disease. For instance, mice with Psmb9 knockout exhibit myelin loss, increased blood-brain barrier permeability, accumulation of amyloid-β, and elevated levels of reactive oxygen species (ROS). Consequently, these factors contribute to chronic oxidative stress, amplified neuroinflammation, and cognitive impairment, thereby stressing the relevance of Lmp2 in preserving optimal brain cell function [28]. The function of Lmp2 in tissue development has been demonstrated in research on neurogenesis. In scenarios with excessive mTORC1 complex activity, the lack of Lmp2 impedes the proliferation of neuronal progenitors, thereby regulating their differentiation [29]. Moreover, the Lmp2 subunit is essential for muscle cell differentiation. Immunoproteasome inhibition in myoblasts causes an increase in oxidized proteins and hinders myoblast differentiation [22].

Given the existing data on the role of Lmp2 in the regulation of cellular proteostasis across different tissue types, it is of particular interest to examine the role played by immunoproteasomes in maintaining ESC pluripotency and differentiation. The distinctive potential of ESCs for self-renewal and differentiation is contingent upon highly regulated processes that underpin genomic stability and proteostasis. The term “pluripotency” denotes the ability of ESCs to selfrenew and differentiate into any cell type within an organism, except for specific extraembryonic tissues, such as the trophoblast and primitive endoderm. Prior to embryo implantation, significant morphological and molecular changes occur in the epiblast, preparing the cells for development. Development of the postimplantation epiblast is preceded by epiblast cell polarization, which is characterized by rosette formation, followed by the formation of the proamniotic cavity through embryo cavitation [31, 32]. During this phase, epiblast cells shift from a state of “naïve” pluripotency to a “primed” state, initiating their differentiation into ecto-, meso-, and endoderm. This transition involves several intermediate states with unique characteristics [33]. To date, at least four distinct types of pluripotent cells with stable *in vitro *culture analogues have been identified [33].

Our prior research has demonstrated that immunoproteasome expression is initiated at the epiblastlike cell stage [15], reaches its highest level on the third day of mesodermal differentiation (unpublished data), and steadily decreases until it becomes undetectable. Epiblast-like cells and cells from the third day of mesodermal differentiation in culture correlate with postimplantation epiblast cells and the primitive streak stage in mouse embryo development. According to public single-cell RNA sequencing (scRNA-seq) data, the peak of immunoproteasome expression also coincides with the stage of primitive streak formation [34, 35].

The formation of the primitive streak involves epiblast cells that undergo an epithelial-mesenchymal transition (EMT) marked by modifications in cell shape due to actin cytoskeleton remodeling, the breakdown of intercellular junctions, degradation of the basement membrane, and the activation of cellextracellular matrix interactions [36]. Furthermore, epiblast cell division proceeds at a notably higher rate during this phase. During the transition to gastrulation, mouse epiblast cells experience a reduction in cell cycle duration from 12 to 14 h to 6 to 8 h [37]. Given the substantial changes characteristic of early embryonic development, ensuring rapid cell adaptation to internal and external signals is critical. Immunoproteasomes are characterized by their rapid assembly kinetics and short half-lives [38]. The hypothesis is that immunoproteasomes may degrade damaged and polyubiquitinated proteins more effectively under conditions of stress and pro-inflammatory signaling (refer to review [11]). It seems safe to assume that the role of immunoproteasomes in early embryonic development is to prevent the accumulation of unnecessary and/or damaged proteins. However, it is worth noting that mice with knockout of all three catalytic immunoproteasome subunits do not exhibit critical neonatal developmental anomalies. This phenomenon may be indicative of compensatory mechanisms that can substitute for their functions. The role of the immunoproteasome in maintaining proteostasis and participating in the differentiation of ESCs is expected to be clarified through studies using cells deprived of both individual catalytic subunits of the immunoproteasome and all three.

## CONCLUSIONS

In conclusion, we have succeeded in generating and characterizing a mouse ESC line with knockout of the* Psmb9 *gene encoding the catalytic subunit of the immunoproteasome Lmp2 (β1i). The absence of Lmp2 was found to have no effect on the morphology, proliferative activity, and pluripotent status of ESCs. This cell line represents a promising tool for investigating the role of the *Psmb9 *gene and immunoproteasomes in the subsequent stages of ESC differentiation* in vitro*.

## Abbreviations

UPS: ubiquitin-proteasome system
ESC: embryonic stem cells
ROS: reactive oxygen species
CL: caspase-like activity
RA: retinoic acid
IFNγ: interferon-γ
